# Ketone body levels in wintering great tits *Parus major* in sites differing in artificial food availability

**DOI:** 10.1093/conphys/coac072

**Published:** 2022-11-24

**Authors:** Adam Kaliński, Michał Glądalski, Marcin Markowski, Joanna Skwarska, Jarosław Wawrzyniak, Jerzy Bańbura

**Affiliations:** Department of Experimental Zoology and Evolutionary Biology, Faculty of Biology and Environmental Protection, University of Łódź, Banacha 12/16, 90-237 Łódź, Poland; Department of Experimental Zoology and Evolutionary Biology, Faculty of Biology and Environmental Protection, University of Łódź, Banacha 12/16, 90-237 Łódź, Poland; Department of Experimental Zoology and Evolutionary Biology, Faculty of Biology and Environmental Protection, University of Łódź, Banacha 12/16, 90-237 Łódź, Poland; Department of Experimental Zoology and Evolutionary Biology, Faculty of Biology and Environmental Protection, University of Łódź, Banacha 12/16, 90-237 Łódź, Poland; Department of Experimental Zoology and Evolutionary Biology, Faculty of Biology and Environmental Protection, University of Łódź, Banacha 12/16, 90-237 Łódź, Poland; Department of Experimental Zoology and Evolutionary Biology, Faculty of Biology and Environmental Protection, University of Łódź, Banacha 12/16, 90-237 Łódź, Poland

**Keywords:** wintering, point-of-care, ketone, great tit

## Abstract

Ketone body levels, among other biochemical blood indices, are important indicators of the physiological condition of birds. Plasma ketone as an indicator of fasting and lipid use is strongly linked to demanding phases in the avian life cycle, such as migration or wintering. The main goal of this study was to check whether ketone body levels differ between the habitats in which individuals stay in winter. To test the above prediction, we used a portable point-of-care device to measure ketone body levels in wintering great tits (*Parus major*). We assumed that wintering in distinct habitats that differ structurally, particularly with respect to food availability, would affect the metabolic performance of birds and their physiological condition. Individual great tits were trapped in mist nets and blood-sampled in three distinct locations within the city: an urban parkland, the deciduous forest and a city centre. As expected, we showed that the mean ketone level was significantly higher in the area where artificial feeding was irregular than in two areas of regular feeding, indicating the more intense fasting state there. We also checked if the level of ketone bodies differs with respect to the sex of an individual, but we found no such difference.

## Introduction

Measuring plasma metabolite levels in wild birds is one of the basic tools in ecophysiological research. Plasma metabolites are products of different biochemical pathways related to energy metabolism and include glucose, cholesterol, triglycerides and ketones, among many others, but also non-energetic resources (Scanes, 2014). Concentrations of these metabolites in the circulating bloodstream indicate the nutritional state of an individua because they change with feeding or fasting (Jenni-Eiermann *et al.*, 2002; Guglielmo *et al*., 2005; Viblanc *et al.*, 2018). The nutritional state of many avian species exhibits daily and seasonal variation because individuals face a variety of internal and external challenges such as food shortages, threat from predation, infestation of ectoparasites and energetically demanding phases such as breeding, migration or wintering (Jenni-Eiermann and Jenni, 1997, 1998). Therefore, studying the metabolic profiles of birds in the wild may provide essential data to understand their physiology during crucial life stages. It is especially important in the context of rapid changes occurring in natural habitats. Many bird populations face habitat fragmentation and destruction and must adapt to a changing climate (Albano, 2012; Mainwaring *et al.*, 2012; Maute *et al.*, 2015; Glądalski *et al.*, 2016). For the above reasons, ecologists and conservationists still seek new and useful devices to effectively assess different indices of physiological body condition in birds under field conditions (Albano, 2012; Minias, 2015).

Ketone bodies, including their most stable form, β-hydroxybutyrate (BOH), are products of fatty acid breakdown in the liver and are metabolized during fasting, reducing protein breakdown (Newman and Verdin, 2014). Ketones are then released into the bloodstream and transported to tissues such as muscles or the brain, where they are converted to acetyl-CoA and used as an energy source (Berg *et al*., 2019). For the above reasons, ketone body concentration provides valuable information on the nutritional state of an individual and may be used as an index of individual physiological condition. Therefore, BOH is used as an index of performance in studies concerning bird migrations, which indicates a metabolic response to long-distance flight (Jenni-Eiermann *et al.*, 2002), stopover performance in refuelling migrants (Guglielmo *et al*., 2005) or mass change during migration (Cerasale and Guglielmo, 2006). In addition, BOH levels can also be used in solving other ecological issues such as responses to weather conditions (Boyle *et al.*, 2010) or may be indicative of ectoparasite infestation (Azeredo *et al.*, 2016).

Despite the advantages in solving the ecological problems resulting from the measurement of ketones in wild bird species, their measurement in the field is challenging. Accurate determination of the level of ketones in samples taken from wild animals has usually been performed in a laboratory using kinetic enzymatic assays (Jenni-Eiermann and Jenni, 1994; Jenni-Eiermann *et al.*, 2002; Viblanc *et al.*, 2018). Collecting and preparing blood samples in the field is a complex procedure that includes centrifugation and freezing, and then transport to the laboratory. The next step, which is the laboratory enzymatic assay of BOH, requires high-precision pipetting of small volumes under a time-restricted work regime, which makes them especially prone to human errors (Sommers *et al.*, 2017). Therefore, portable ketone measuring devices offer an alternative to laboratory tests and allow quick and reliable measurements to be obtained at a relatively low cost and with little blood. The devices have already been used in the case of domestic animals such as sheep or dairy cows (Hornig *et al.*, 2013; Geishauser *et al.*, 2001; Iwersen *et al*., 2013; Xu *et al.*, 2017) and have also been used successfully in wild bat species (Boyles *et al.*, 2016). However, portable devices have rarely been used to measure ketones in wild birds and we are only aware of three studies so far (Azeredo *et al.*, 2016; Sommers *et al.*, 2017; Lindholm *et al.*, 2019).

Lindholm *et al.* (2019) validated a point-of-care (POC) device for use in birds and found strong agreement of the obtained measurements of the levels of ketone bodies between the device and the widely used standard laboratory methodology. Lindholm *et al.* (2019) studied indoor-raised red junglefowl (*Gallus gallus*), kept under a strict feeding regime. Since their study validated the application of the device in the assessment of ketone bodies in avian species, we decided in our study to measure blood ketone concentrations by using the same type of POC device in wild species, wintering great tit (*Parus major*). The great tit is a small, partly migratory passerine (Gosler *et al.*, 2020), for which the winter season is a challenging period, particularly for migrating individuals. We assume that the degree of fasting and the use of fatty acids in this species may be related to the food availability of the environment where birds stay for shorter or longer periods during winter, including the availability of human-provided food. Relatively poor feeding conditions resulting from the irregular provision of artificial food can trigger the utilization of fatty acids to a greater extent than richer conditions resulting from the regular provision, regardless of habitat. Therefore, we suppose that the extensive parkland area with sparse tree and bush cover where artificial food is provided irregularly offers relatively poorer feeding conditions during winter compared to the forest and city centre sites where artificial food is provided regularly. For the above reasons, we expected variation in blood ketone body levels in individuals wintering in the three locations, differing with respect to the physical structure of a particular habitat and most important to its food availability. We expected that the variation of the study sites in the prevailing feeding conditions would allow the corresponding differences to be found in the level of ketone bodies detectable with the POC device.

**Figure 1:**
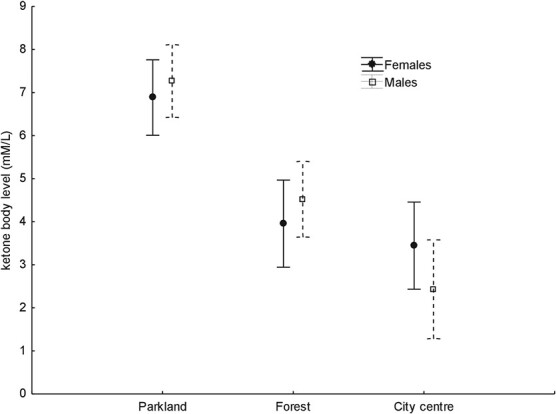
Mean (± standard errors) in females and males ketone body levels of great tit in the three study areas.

## Methods

The study was carried out during one winter season (2019/2020). The study areas were winter bird feeding stations placed around the city of Łódź in the three different locations: an urban park, a deciduous forest and a highly urbanized fragment of the city centre. The parkland study site (51°45’N; 19°24′E) is located in the SW part of Łódź in the Botanical Garden, which is characterized by a fragmented tree cover and a high proportion of alien plant species. The south, southeast and north sides of the Botanical Garden border the highly urbanized area of the town with numerous blocks of flats. Regular visitors are not allowed in the garden during winter. The feeding station in the botanical garden was located close to its western border with an adjacent area of wastelands covered mainly by low bushes and low herbal vegetation. This feeding station was provided with food 2 days before the day of trapping birds.

At the forest study site, our feeding site is located next to one of the buildings (Ecological Education Centre) in a forest clearing along the road that crosses the Łagiewniki Forest (51°50′N; 19°29′E). In the clearing there is also a hospital and a wildlife rescue centre, where several regular feeding stations operate. The total area of this deciduous forest is ~1250 ha, neighbouring the NE suburbia of Łódź. The dominant tree species in the forest are *Quercus robur* and *Q. petraea* oaks.

The city centre study site (51°46′N; 19°29′E) Łódź is located next to the main building of the Faculty of Biology and Environmental Protection of the University of Łódź and is surrounded by tall buildings, sidewalks, parking, streets with heavy traffic and relatively little space covered with trees and bushes. There are many permanent feeding stations operating in this area. The quantity of feeders was assessed in each study site by counting them within ~0.8 km radius from the trapping sites. We found no other feeding sites in the Botanical Garden, 6 and 10 additional feeding sites in the forest study site and at the city centre, respectively.

During the study, birds were captured in a standardized manner in ornithological mist nets. Two 5-m-long mist nets positioned fixedly during each trapping were used in the three study areas. Birds were captured twice in each study area: once in December 2019 and once in the following month (20th December and 9th January in the parkland, 5th December and 16th January in the forest, 4th December and 27th January in the city centre). Two days before each trapping, a firm amount of sunflower seeds was placed at each site in standardized bird feeders. Additionally, during each trapping session, specific song playbacks were used to attract birds. During the study, 62 individuals (30 females: 12 in the parkland, 9 in the forest, 9 in the city centre; 32 males: 13 in the parkland, 12 in the forest, 7 in the city centre) were sampled. All individuals were captured within a 4-hour period during the day (9.00–13.00) to avoid possible fluctuations in ketone levels due to the diurnal schedules of wintering birds. All individuals were ringed, weighed to the nearest 0.1 g using an electronic scale and their sex was determined (Jenni and Winkler, 1994). Subsequently, each individual was bled from the ulnar vein and a sample of ~1.5 μl was taken. Blood samples were taken immediately after banding to avoid potential stress responses in birds. The samples were moved to test strips and analyzed on a portable POC device (Optium Xido Neo, Abbott, IL, USA) to measure BOH levels (mM/l). The birds were released immediately after sampling. As described by Lindholm *et al.* (2019), the device overestimated ketone levels compared to laboratory-derived values; nevertheless, laboratory vs. device values correlated well, indicating that the Optium Xido Neo is accurate enough and to be used in field studies. In nine cases in the parkland area values higher than the device measurement range (0.0–8.0 mM/l), we assumed a value of 8.1 mM/l for calculations. The distribution of body ketone values obtained for both sexes at the three study sites is presented in Fig. 1.

### Weather Conditions

The winter season of 2019/2020 was characterized by mild weather conditions with average temperatures in December and January above 0°C. On bird trapping days the average temperatures were also above 0°C ranging from 0°C to 7.8°C. Precipitation was at relatively low levels, with rain dominating over snowfall. The sum of rain and snowmelt in December 2019 and January 2020 was 26.4 mm and 29.5 mm, respectively.

### Statistical Analysis

The ketone concentration was analyzed using general linear models. It was tested whether the study area and sex affected the concentration of ketones. Sex and the study area were included as fixed factors in this model, both as main effects and as interactions with each other. Since we expected that body mass and sampling order may covary with ketone concentrations, we added these traits as covariates. This analysis was performed with the concentration of BOH as the dependent variable (Crawley, 2002). The full model was simplified by removing non-significant interactions beginning from the least significant ones to obtain a final model that included all main effects (Crawley, 2002). Since only in the parkland area some values of the ketone concentration exceeded the measurement range, we added a separate validation of our approach. We performed χ^2^ test of independence to examine how the two categories of ketone concentration (≤8.0 mM/l vs. >8.0) are related to the three study sites (2 × 3 contingency table). In addition, a simple general linear model was applied to compare body mass between the three sites and between the two sexes. Linear modeling was performed using IBM SPSS Statistics 22 software (Heck *et al.*, 2010; IBM SPSS Statistics 22, 2013).

**Table 1:** Summary of the general linear model for the concentrations of the ketone body (mM/l). The effects of sex and study area and the interaction between the main factors and body weight and sampling order as covariates are given

Model/factor	Df	F	*P*
Final model			
Intercept	1; 52	14.338	**0.015**
Sex	1; 52	0.737	0.395
Study area	2; 52	28.026	**<0.001**
Body weight	1;52	1.867	0.178
Sampling order	1;52	1.060	0.308
Removed interactions			
Study area^*^sex	2;52	1.976	0.149
Sex^*^sampling order	1;52	0.588	0.477
Sex^*^body weight	1;52	0.910	0.344

## Results

Mean blood ketone levels differed significantly between study areas and were highest in the parkland study area with no significant differences between the forest study areas and the city centre as well as between females and males (Table 1, Fig. 1). Both body weight and sampling order covariates did not affect ketone levels in males or females (Table 1). χ^2^ test of independence (χ^2^_2_ = 15.40; *P* < 0.001) showed that the parkland study site differs distinctly from both the forest and city centre study sites with respect to the distribution of the values exceeding the measurement range of the device applied, which confirms the result of the general linear model (Table 1, Fig. 1).

**Figure 2:**
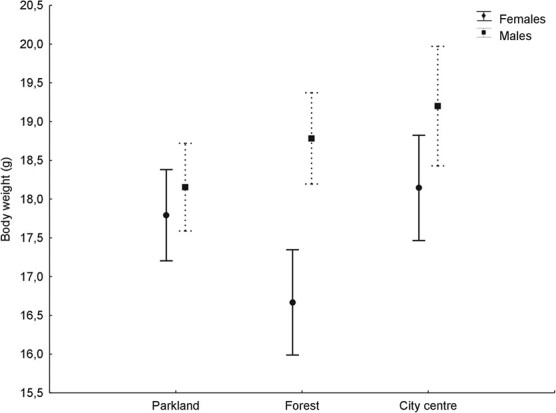
Mean (± standard errors) body weight in females and males of great tit in the three study areas.

The body mass of the individuals sampled differed significantly between the three study sites for both sexes—on average, highest in the city centre area (Table 2, Fig. 2).

**Table 2:** Summary of the two-way general linear model of body weight for female and male great tits across the three study sites

Factor	Df	F	*P*
Study area	2; 56	4.05	**0.022**
Sex	1; 56	19.82	**<0.001**
Study area^*^Sex	2;56	4.20	**0.020**

## Discussion

We used a handheld POC device to measure the levels of ketone bodies in a small passerine species, the great tit, in the winter season. As expected, we found a considerable variation within the trait with the highest mean ketone concentrations in the parkland area. Since plasma BOH is indicative of fasting and lipid use, our results suggest that the trophic conditions for wintering tits were relatively poorer at the parkland study site.

The use of portable devices to assess biochemical parameters gave researchers new tools in the field of ecology. In their review paper, Lindholm and Altimiras (2016) have shown that avian physiologists require portable and accurate devices for use under field conditions. Initially, handheld devices, designed specifically for human diagnostics (Price, 2001), were validated in domestic animals (Hornig *et al.*, 2013; Iwersen *et al.*, 2013; Bach *et al.*, 2016), and at present various types of analyzers are used in veterinary practice. In general, these studies show that POC meters provide reliable BOH results in mammals and birds, though absolute values may not be comparable between methods. The successful application of these devices in domestic animals has led to the use of POC in wild avian species. Azeredo *et al.* (2016) in the first such study verified correlations between ketone bodies and different ecological variables in pale-bellied tyrant-manakin (*Neopelma pallescens*). They have shown that ketone levels were higher in the morning, suggesting a fasting period during the night. They have also shown that a higher infestation positively correlated with ketone levels. The first study in which a handheld ketone meter was compared with a laboratory enzymatic assay was conducted by Sommers *et al.* (2017). Blood samples were obtained from individuals of grasshopper sparrow (*Ammodramus savannarum*). The authors found that the measures were precise and as accurate as the laboratory assay method. However, the device they used is of limited applicability in wild avian species as its upper measurement range is 2 mM/l. Earlier studies using kinetic assays showed that prolonged fasting may lead to BOH concentrations exceeding this value, for example, in king penguin (*Aptenodytes patagonicus*) chicks (Cherel and Le Maho, 1985) or in herring gulls (*Larus argentatus*) where the values were as high as 8.2 mM/l (Totzke *et al.*, 1999). Therefore, Lindholm *et al.* (2019) used a POC ketone meter with an upper measure limit of 8.0 mM/l. They studied intermittently fed red junglefowl and ascertained a rapid increase in BOH concentrations to more than 6 mM/l after 40 h of fasting. These high levels of ketone bodies decreased after 4 h of refeeding, indicating a return to glycolytic metabolism.

Following the method used by Lindholm *et al.* (2019), we studied BOH levels in wintering great tits to assess the degree of fasting during a demanding period of life cycle. Ketone bodies are essential in energy metabolism during fasting, as they allow birds to survive by mobilizing fatty acids, mainly from subcutaneous fat deposits. There is a marked diurnal variation in body mass in small wintering passerines, since after dusk birds cannot forage and are forced to fast during the night hours (Liliendahl *et al.*, 1996; Liliendahl, 2002). Therefore, individuals are capable of depositing during the day 10% of their morning body mass as fat reserves for the coming night (Haftorn, 1992). Krams *et al.* (2013) showed that during nights with temperatures far below zero, surviving individuals lost on average 12.78% of their evening body mass with values twice as high in individuals which did not survive the night. The main factor reducing the time available for foraging in high latitudes is the short period of daylight accompanied by restricted access to food due to snow and ice cover (Krams *et al.*, 2010). However, the diurnal patterns in foraging activity and mass gain in tits are also shaped by predation risk, and a trade-off between starvation and predation risk appears (Lima, 1986; Witter and Cuthill, 1993; Bonter *et al.*, 2013). The hypothesis predicts that with increasing predation risk, subcutaneous fat reserves should decrease (Gosler *et al.*, 1995; Krams, 2000). Moiron *et al.* (2018) showed that great tits in the studied population demonstrated substantial variation in body mass and foraging strategies. However, their findings implied that the increased energetic demands experienced by tits in winter could favour individuals that prioritize avoiding the risk of starvation rather than maximizing predation avoidance. However, we should stress here that we do not have data on predation rates in our study sites but our study scheme suggest that the risk of predation is generally low at all study sites. Furthermore, in dominance-structured flocks of wintering tits, dominant individuals may take their advantage over subordinates of their priority to food resources and lower the risk of starvation (Verhulst and Hogstad, 1996; Krams, 2000; Krams *et al.*, 2013). For the reasons mentioned above, we measured BOH levels in great tits wintering under different environmental conditions and found that great tits were characterized by, on average, higher ketone concentrations in the parkland study area than in both the forest and the city centre.

Our results suggest that individuals in the parkland faced the relatively worst feeding conditions compared to the forest and the city centre. We suppose that the pattern we found results mainly from availability and distribution of a novel, superabundant food source for wintering passerines in the form of feeders supplied with seeds or nuts, since it is well known that great tits are among species readily using feeders (Chamberlain *et al.*, 2005; own observations). Such feeders have recently become a regular feature of urban landscapes throughout the northern hemisphere (Galbraith *et al.*, 2017; Tryjanowski *et al.*, 2017). In our city centre study site feeders are very common with 10 of them fixed to the windows in the building of our faculty, not counting at least a few of them in the nearby residential area. Similarly, the forest study site, which is located in a kind of artificial clearing surrounded by a coherent, uniform woodland area, is a place where six bird feeders operate in all three institutions located there. What is of crucial importance in the context of this study is that most of these feeders are regularly supplemented with fresh seeds, which makes them a stable food source during winter. In contrast to the city centre and forest sites, the feeders are sparsely located on the edges of the botanical garden, mainly in a residential area adjoining the garden from the east (own observations). It creates an extensive parkland area with relatively sparsely located feeders. Furthermore, the density of wintering individuals, which is relatively high in parkland but not in the forest and city (own observations), intensifies competition for food. We suppose that it may result from a large number of long-distant migrating individuals attracted by a vast green parkland area as a potentially good stopover site. If this assumption is true, it is in line with our interpretation, since long-distance migrants rely mainly on their fat reserves as an energy source (Jenni-Eiermann *et al.*, 2002). Therefore, it is possible that the parkland area in winter may act as a kind of ecological trap for migrating tits, which attracts them but offers a relatively small number of stable food sources. Nevertheless, we do not know the proportion of migrants to residents during winter in our study sites, but on the basis of bird ringing data during the breeding period we suppose that almost all individuals sampled in this study are migrants. Although we believe that the presence and distribution of feeders are key factors in the context of this study, other factors, such as predation, cannot be excluded. Our main result is also supported by the analysis of the body mass of sampled individuals, which shows that the heaviest individuals were sampled at the city centre site, though this effect was clearer in the case of males. Interestingly, the body mass of females in the forest is lowest compared to all other groups without accompanying increase in ketone concentration. It suggests that the relationship between body mass and lipid metabolism in wintering tits is not always straightforward. Other unknown factors may play a role in this case.

In conclusion, we should emphasize that the portable device we used in this study to measure ketones in the field has already been successfully validated in a carefully designed study (Lindholm *et al.*, 2019). The authors recommended that ketone body POC measures be part of bird assessment of the body condition for researchers working in the field. Our results support their findings and show that this method provides reliable data on one of the key features of avian metabolism. As expected, circulating ketone levels were on average higher in the environment, which offers the relatively poorest conditions for wintering birds. Therefore, we support the conclusion that the Optium Xido Neo measuring system offers reliable results at lower costs than laboratory assays methods, a lower level of expertise and lower blood volumes required. However, it should be remembered that the measurement range of the device may be inadequate in cases of high ketosis. Therefore, in such cases, we recommend a simple procedure consisting of dilution samples 1:1. Nevertheless, we recommend using this measuring system with care for scientists working directly in the field on wild bird populations. Our results suggest that the POC device we used may deliver data that indirectly indicate the quality of environments in terms of food availability; therefore, we think that this tool may be useful for conservationists seeking simple to obtain and reliable indexes of habitat quality.

## Funding

This work was supported by own funds of the University of Łódź.

## Conflict of interest

The authors declare that they have no conflict of interest.

## References

[ref1] Albano N (2012) Conservation physiology tools: their role in assessing habitat quality in birds. Ardeola 59: 197–216. 10.13157/arla.59.2.2012.197.

[ref2] Azeredo LMM , Oliveira TC, Lopez LCS (2016) Blood metabolites as predictors to evaluate the body condition of *Neopelma pallescens* (Paseriformes: *Pipiridae*) in northeastern Brazil. Fortschr Zool 33: 1–9.

[ref3] Bach KD , Heuwieser M, McArt JAA (2016) Technical note: comparise of 4 electronic handheld meters for diagnosing hyperketonemia in dairy cows. J Dairy Sci 99: 9136–9142. 10.3168/jds.2016-11077. 27568045

[ref4] Berg JM , Tymoczko JJ, Stryer L (2019) Biochemistry. Freeman, New York, pp. 2096–2099

[ref5] Bonter DN , Zuckerberg B, Sedgwick CW, Hochachka WM (2013) Daily foraging patterns in free-living birds: exploring the predation-starvation trade-off. Proc R Soc B 280: 20123087. 10.1098/rspb.2012.3087. PMC3652453 23595267

[ref6] Boyle WA , Norris DR, Guglielmo CG (2010) Storms drive altitudinal migration in a tropical bird. Proc R Soc B 277: 2511–2519. 10.1098/rspb.2010.0344. PMC2894928 20375047

[ref7] Boyles JG , McGuire LP, Boyles E, Reimer JP, Brooks CAC, Rutherford RW, Rutherford TA, Whitaker JO, McCracken GF (2016) Physiological and behavioral adaptations in bats living at high latitudes. Physiol Behav 165: 322–327. 10.1016/j.physbeh.2016.08.016. 27542518

[ref8] Cerasale DJ , Guglielmo CG (2006) Dietary effects on prediction of body mass changes in birds by plasma metabolites. Auk 123: 836–846. 10.1093/auk/123.3.836.

[ref9] Chamberlain DE , Vickery JA, Glue DE, Robinson RA, Conway GJ, Woodburn RJ, Cannon AR (2005) Annual and seasonal trends in the use of garden feeders by birds in winter. Ibis 147: 563–575. 10.1111/j.1474-919x.2005.00430.x.

[ref10] Cherel Y , Le Maho Y (1985) Five months of fasting in king penguin chicks: body mass loss and fuel metabolism. Am J Physiol 249: R387–R392. 10.1152/ajpregu.1985.249.4.R387. 4051024 10.1152/ajpregu.1985.249.4.R387

[ref11] Crawley MJ (2002) Statistical computing: an introduction to data analysis using S-Plus. Wiley, Chichester

[ref12] Galbraith JA , Jones DN, Beggs JR, Parry K, Stanley MC (2017) Urban bird feeders dominated by a few species and individuals. Front Ecol Evol 5: 81. 10.3389/fevo.2017.00081.

[ref13] Geishauser T , Leslie K, Kelton D, Duffield T (2001) Monitoring for subclinical ketosis in dairy herds. Compend Contin Educ Pract Vet 23: S65–S71.

[ref14] Glądalski M , Bańbura M, Kaliński A, Markowski M, Skwarska J, Wawrzyniak J, Zieliński P, Bańbura J (2016) Effects of extreme thermal conditions on plasticity in breeding phenology and double-broodedness of great tits and blue tits in Central Poland in 2013 and 2014. Int J Biometeorol 60: 1795–1800. 10.1007/s00484-016-1152-9. 26983847 PMC5085981

[ref15] Gosler A , Clement P, Christie DA (2020) *Great Tit* (*Parus major*), version 1.0. In S. M. Billerman, B. K. Keeney, P. G. Rodewald, and T. S. Schulenberg, eds, Birds of the World. Cornell Lab of Ornithology, Ithaca, NY, USA. 10.2173/bow.gretit1.01

[ref16] Gosler AG , Greenwood JJD, Perrins C (1995) Predation risk and the cost of being fat. Nature 377: 621–623. 10.1038/377621a0.

[ref17] Guglielmo CG , Cerasale DJ, Eldermire C (2005) A field validation of plasma metabolite profiling to assess refuelling performance of migratory birds. Physiol Biochem Zool 78: 116–125. 10.1086/425198. 15702470

[ref19] Haftorn S (1992) The diurnal body weight cycle in titmice *Parus* spp. Ornis Scand 23: 435–443. 10.2307/3676674.

[ref20] Heck RH , Thomas SL, Tabata LN (2010) Multilevel and Longitudinal Modeling with IBM SPSS. Routledge, New York.

[ref21] Hornig KJ , Byers SR, Callan RJ, Holt T, Field M, Han H (2013) Evaluation of a point-of-care glucose and beta-hydroxybutyrate meter operated in various environmental conditions in prepartum and postpartum sheep. Am J Vet Res 74: 1059–1065. 10.2460/ajvr.74.8.1059. 23879842

[ref22] IBM SPSS Statistics 22 (2013) SPSS for WindowsRelease 22.0. IBM Corporation

[ref23] Iwersen M , Klein-Jobstl D, Pichler M, Roland R, Fidlschuster B, Schwandenwein I, Drillich M (2013) Comparison of 2 electronic cowside tests to detect subclinical ketosis in dairy cows and the influence of the temperature and type of blood sample on the test results. J Dairy Sci 96: 7719–7730. 10.3168/jds.2013-7121. 24140315

[ref24] Jenni L , Winkler R (1994) Moult and ageing of European passerines. Academic Press, London, pp. 150–151

[ref25] Jenni-Eiermann S , Jenni L (1994) Plasma metabolite levels predict individual body-mass changes in a small long-distance migrant, the garden warbler. Auk 111: 888–899. 10.2307/4088821.

[ref26] Jenni-Eiermann S , Jenni L (1997) Diurnal variation of metabolic responses to short-term fasting in passerine birds during the postbreeding, molting and migratory period. Condor 99: 113–122. 10.2307/1370229.

[ref27] Jenni-Eiermann S , Jenni L (1998) What can plasma metabolites tell us about the metabolism, physiological state and condition of individual bird? An overview. Biol Cons Fauna 102: 312–319.

[ref28] Jenni-Eiermann S , Jenni L, Lindström KA, Piersma T, Visser GH (2002) Fuel use and metabolic response to endurance exercise: a wind tunnel study of a long-distance migrant shorebirds. J Exp Biol 205: 2453–2460. 10.1242/jeb.205.16.2453. 12124368

[ref29] Krams I (2000) Length of feeding day and body weight of great tits in a single- and a two-predator environment. Behav Ecol Sociobiol 48: 147–153. 10.1007/s002650000214.

[ref30] Krams I , Cirule D, Suraka V, Krams T, Rantala MJ, Ramey G (2010) Fattening strategies of wintering great tits support the optimal body mass hypothesis under condition of extremely low ambient temperature. Funct Ecol 24: 172–177. 10.1111/j.1365-2435.2009.01628.x.

[ref31] Krams I , Cirule D, Vrublevska J, Nord A, Rantala MJ, Krama T (2013) Nocturnal loss of body reserves reveals high survival risk for subordinate great tits wintering at extremely low ambient temperatures. Oecologia 172: 339–346. 10.1007/s00442-012-2505-7. 23086507

[ref32] Liliendahl K (2002) Daily patterns of body mass gain in four species of small wintering birds. J Avian Biol 33: 212–218. 10.1034/j.1600-048X.2002.330302.x.

[ref33] Liliendahl K , Carlson A, Welander J, Ekman JB (1996) Behavioural control of daily fattening in great tits (*Parus major*). Can J Zool 74: 1612–1616. 10.1139/z96-178.

[ref34] Lima SL (1986) Predation risk and unpredictable feeding conditions: determinants in body mass in birds. Ecology 67: 377–385. 10.2307/1938580.

[ref35] Lindholm C , Altimiras J (2016) Point-of-care devices for physiological measurements in field conditions. A smorgasbord of instruments and validation procedures. Comp Biochem Phys A 202: 99–111. 10.1016/j.cbpa.2016.04.009. 27083239

[ref36] Lindholm C , Altimiras J, Lees J (2019) Measuring ketones in the field: rapid and reliable measures of β-hydroxybutyrate in birds. Ibis 161: 205–210. 10.1111/ibi.12643.

[ref37] Mainwaring MC , Hartley IR, Bearhop S, Brulez K, du Feu CR, Murphy G, Plummer KE, Webber SL, Reynolds JS, Deeming DC (2012) Latitudinal variation in blue tit and great tit nest characteristics indicates environmental adjustment. J Biogeogr 39: 1669–1677. 10.1111/j.1365-2699.2012.02724.x.

[ref38] Maute K , French K, Legge S, Astheimer L, Garnett S (2015) Condition index monitoring supports conservation priorities for the protection of threatened grass-finch populations. Conserv Physiol 3: 1–9. 10.1093/conphys/cov025. PMC4778451 27293710

[ref39] Minias P (2015) The use of haemoglobin concentrations to assess physiological condition in birds: a review. Conserv Phys Ther 3: cov007. 10.1093/conphys/cov007 PMC4778452 27293692

[ref40] Moiron M , Mathot KJ, Dingemanse NJ (2018) To eat and not to be eaten: diurnal mass gain and foraging strategies in wintering great tits. Proc R Soc B 285: 1–10. 10.1098/rspb.2017.2868. PMC5879632 29540518

[ref41] Newman JC , Verdin E (2014) Ketone bodies as signalling metabolites. Trends Endocrinol Metab 25: 42–52. 10.1016/j.tem.2013.09.002. 24140022 PMC4176946

[ref42] Price CP (2001) Point of care testing. BMJ 322: 1285–1288. 10.1136/bmj.322.7297.1285. 11375233 PMC1120384

[ref43] Scanes CG (2014) Sturkie’s Avian Physiology. Academic Press, London, pp. 421–451.

[ref44] Sommers AS , Boyle AW, McGuire LP (2017) Validation of a field-ready handheld meter for plasma β-hydroxybutyrate analysis. J Field Ornithol 88: 399–404. 10.1111/jofo.12233.

[ref45] Totzke U , Fenske M, Huppop O, Raabe H, Schach N (1999) The influence of fasting on blood and plasma composition of herring gulls (*Larus argentatus*). Physiol Biochem Zool 72: 426–437. 10.1086/316675. 10438680

[ref46] Tryjanowski P , Skórka P, Møller AP (2017) Intra- and interspecific abundance of birds affects detection of novel food sources by great tits Parus major. Acta Orn 52: 221–231. 10.3161/00016454AO2017.52.2.009.

[ref47] Verhulst S , Hogstad O (1996) Social dominance and energy reserves in flocks of willow tits. J Avian Biol 27: 203–208. 10.2307/3677223.

[ref48] Viblanc VA , Schull Q, Cornioley T, Stier A, Menard J-J, Groscolas R, Robin J-P (2018) An integrative appraisal of the hormonal and metabolic changes induced by acute stress using king penguins as a model. Gen Comp Endocrinol 269: 1–10. 10.1016/j.ygcen.2017.08.024. 28843614

[ref49] Witter MS , Cuthill IC (1993) The ecological costs of avian fat storage. Phil Trans R Soc Lond B 340: 73–92. 10.1098/rstb.1993.0050. 8099746

[ref50] Xu S , Wu Z, Zou Y, Li S, Cao Z (2017) Evaluation of a hand-held meter to detect subclinical ketosis in dairy cows. Adv Dairy Res 05: 1–4. 10.4172/2329-888X.1000173.

